# Catheter-Based Renal Denervation in Uncontrolled and Resistant Hypertension: Evidence, Guidelines, Special Populations and Clinical Implementation

**DOI:** 10.3390/jcdd13090443

**Published:** 2026-09-07

**Authors:** Ioannis Skalidis, Leonidas Koliastasis, Panayotis K. Vlachakis, Anastasios Apostolos, Maria Bozika, Kassiani-Maria Nastouli, Nicolas Amabile, Philippe Garot, Thomas Hovasse, Mariama Akodad, Livio D’Angelo, Antoinette Neylon, Thierry Unterseeh, Roswitha Schreier, Giacomo Maria Cioffi, Julius Jelisejevas, Peter Wenaweser, Pascal Meier, Mario Togni, Stephane Cook

**Affiliations:** 1 Department of Cardiology, HFR—Fribourg Cantonal Hospital and University, 1708 Fribourg, Switzerland; 2 Unit of Structural and Valvular Heart Diseases, First Department of Cardiology, National and Kapodestrian University of Athens, General Hospital of Athens “Hippokration”, 11527 Athens, Greece; 3 Department of Cardiology, Guy’s and St Thomas’ NHS Foundation Trust, Harefield Hospital, London UB9 6JH, UK; 4 School of Medicine, University of Patras, 26504 Patras, Greece; 5 Ramsay Santé, Institut Cardiovasculaire Paris-Sud, Hôpital Jacques Cartier, 91300 Massy, France

**Keywords:** renal denervation, resistant hypertension, uncontrolled hypertension, sympathetic nervous system, ambulatory blood pressure monitoring, device-based therapy

## Abstract

Hypertension remains the leading modifiable cause of cardiovascular death, yet fewer than one in four treated adults achieves guideline-recommended blood pressure targets. Catheter-based renal denervation interrupts efferent and afferent renal sympathetic signalling and lowers blood pressure continuously and independently of medication adherence. After a decade of controversy following the neutral SYMPLICITY HTN-3 trial, a coherent body of second-generation sham-controlled randomised evidence, generated with radiofrequency, endovascular ultrasound, and perivascular alcohol platforms, has established a reproducible, modest, and durable antihypertensive effect with a favourable safety profile. Between 2024 and 2026, the field crossed three thresholds simultaneously: guideline endorsement on both sides of the Atlantic, including the first United States recommendation in the 2025 AHA/ACC guideline; a national coverage determination with coverage with evidence development; and the maturation of randomised and real-world follow-up to three years in cohorts exceeding 3000 patients. This state-of-the-art review synthesises the contemporary evidence base; quantifies the expected treatment effect and its heterogeneity; appraises safety across renal and vascular domains; examines emerging data in chronic kidney disease, diabetes, heart failure, and atrial fibrillation; and details the practical requirements for delivering renal denervation responsibly. We conclude by defining the research agenda that must now be addressed: the identification of responders, the development of an intraprocedural endpoint, a direct comparison with newly approved pharmacotherapy, and evidence of cardiovascular event reduction.

## 1. Introduction

Elevated blood pressure (BP) is the leading modifiable contributor to global cardiovascular morbidity and mortality, affecting approximately 1.3 billion adults worldwide [1]. In the United States, hypertension affects roughly 48% of adults, yet only about one in five achieve the guideline-recommended target of below 130/80 mm Hg [2]. Prevalence rises steeply with age, exceeding 75% beyond 65 years, and the clinical phenotype becomes progressively more complex as multimorbidity accumulates. The consequences are graded and continuous: in an analysis of more than 36 million BP measurements from 1.3 million adults, each incremental rise in systolic BP (SBP) above 130 mm Hg conferred an approximately 18% higher risk of myocardial infarction or stroke over eight years [3].

This gap is not primarily a gap in pharmacology. Effective, inexpensive, and well-tolerated drug classes have been available for decades. The gap is one of implementation and persistence, arising from medication non-adherence that approaches 50% even under the scrutiny of clinical trials, from therapeutic inertia, intolerance, and adverse effects, as well as, in perhaps 10% of treated patients, from true resistance to a three-drug regimen including a diuretic [4]. A therapy that lowers BP continuously and independently of daily patient behaviour therefore addresses a mechanistically distinct problem from adding a further tablet.

Catheter-based renal denervation (RDN) is that therapy. Its trajectory over 15 years, from spectacular uncontrolled results, through a definitive neutral sham-controlled trial, to methodological reconstruction and eventual regulatory approval, is among the most instructive case studies in device-based cardiovascular medicine. The purpose of this review is to describe where the field stands in mid-2026: what the evidence now supports with confidence, what it does not, and what the practising interventional cardiologist must have in place to deliver RDN responsibly.

For this narrative review, the literature was identified through searches of PubMed/MEDLINE and Google Scholar for publications available through July 2026, supplemented by relevant guideline, regulatory, and trial-registry sources. Search terms included combinations of “renal denervation,” “renal sympathetic denervation,” “hypertension,” “resistant hypertension,” and “uncontrolled hypertension.” Priority was given to randomised controlled trials, meta-analyses, large prospective registries, contemporary clinical guidelines, and regulatory documents, as well as studies providing relevant mechanistic, safety, or long-term follow-up data. References of key publications were also screened to identify additional relevant studies.

## 2. Pathophysiological Rationale and Renal Nerve Anatomy

**Figure 1:**
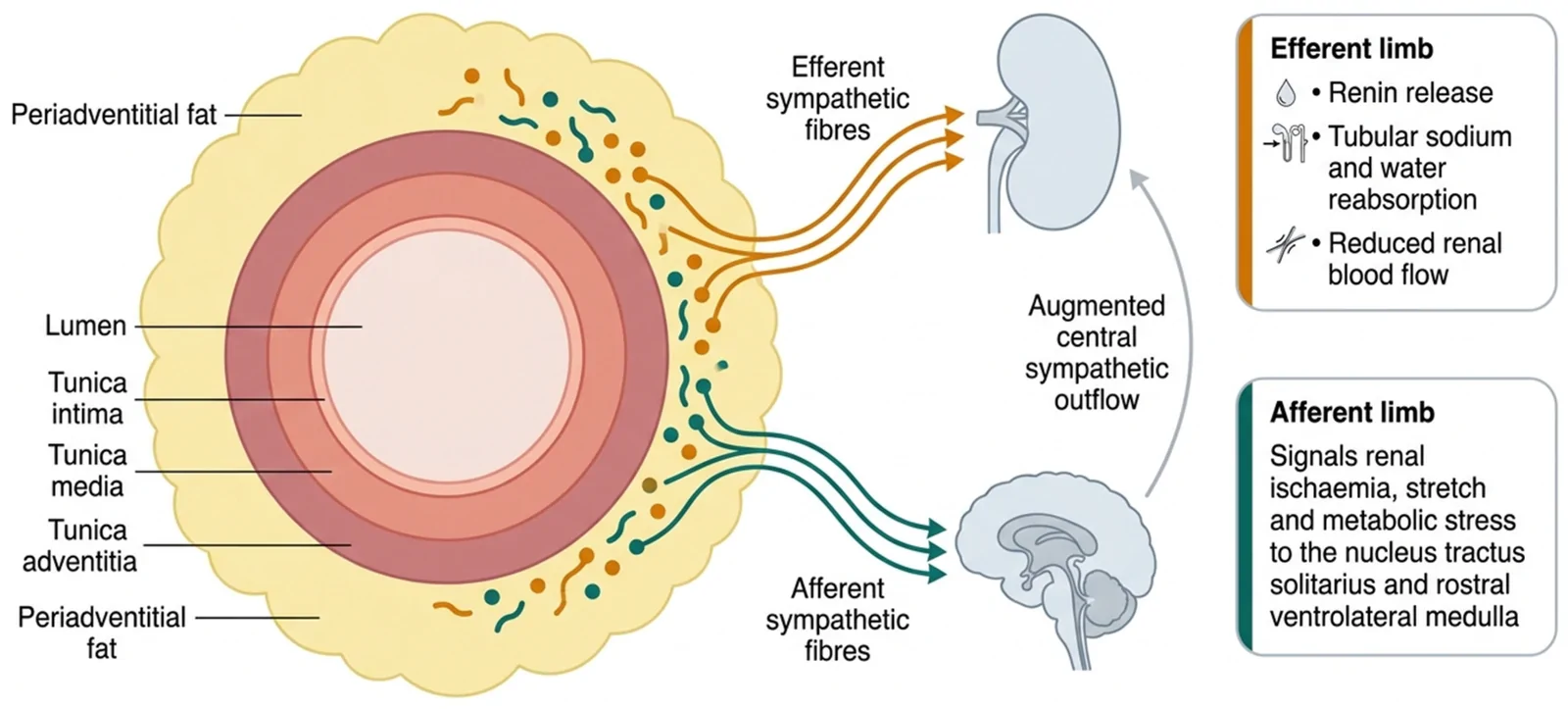
Efferent and afferent renal sympathetic pathways in hypertension. Efferent renal sympathetic fibres stimulate renin release, increase tubular sodium and water reabsorption, and reduce renal blood flow. Renal afferent sensory fibres transmit signals generated by renal ischaemia, mechanical stretch, and metabolic stress to central autonomic nuclei, including the nucleus tractus solitarius and rostral ventrolateral medulla, thereby augmenting central sympathetic outflow. Catheter-based renal denervation targets periarterial renal nerves within the adventitial and periadventitial tissues and may interrupt both neural pathways.

The kidney is both an effector and an origin of sympathetic activity. Efferent renal sympathetic fibres stimulate renin release from the juxtaglomerular apparatus, promote tubular sodium and water reabsorption, and reduce renal blood flow, thereby activating the renin–angiotensin–aldosterone system and expanding the intravascular volume. Afferent fibres, arising from renal mechanoreceptors and chemoreceptors, relay signals of renal ischaemia, stretch, and metabolic stress to the nucleus tractus solitarius and rostral ventrolateral medulla, amplifying central sympathetic outflow to the heart, vasculature, and contralateral kidney [5,6]. RDN interrupts both limbs and, in humans, reduces renal noradrenaline spillover and plasma renin activity [5] (Figure 1).

Two consequences follow that are clinically distinctive. First, the effect is continuous. Blood pressure is lowered across the full 24 h cycle, including the vulnerable early-morning period during which the pharmacodynamic effect of even correctly taken once-daily agents is waning; this is reflected in the consistent reduction of nighttime BP across trials, the ambulatory metric most tightly linked to cardiovascular outcome [6]. Second, the effect is independent of adherence, which is precisely the failure mode that limits pharmacotherapy in the target population.

An important caveat deserves statement. Microneurographic studies of muscle sympathetic nerve activity after RDN have been inconsistent, and the temporal profiles of BP reduction, which is early, and of measurable sympathoinhibition, which is late, do not always align. The causal chain from denervation to BP reduction may therefore be less direct than the mechanistic schematic implies. The BP effect is empirically robust; the mechanism is less completely characterised than is usually acknowledged.

Human histological and microdissection studies transformed procedural practice. Nerve density and proximity to the lumen increase distally, such that 94% of nerves lie within 3 mm of the lumen distal to the first renal artery bifurcation compared with 46% in the proximal segment [7,8]. Approximately 63% of kidneys have late-arriving nerves that join the vascular bundle only beyond the bifurcation, and around 30% of individuals have at least one accessory renal artery [8,9]. First-generation devices ablated the main renal artery only, from a single electrode, with a mean of roughly 11 ablations per patient, a strategy now understood to have been anatomically inadequate. Contemporary radiofrequency protocols with the SYMPLICITY Spyral system target the main renal artery, suitable first-order branches, and accessory renal arteries of 3 to 8 mm in diameter, delivering 20 to 48 ablations per patient [10].

## 3. Device Platforms and Procedural Techniques

Two systems hold United States Food and Drug Administration approval: the Paradise endovascular ultrasound system (Recor Medical), approved on 7 November 2023, and the SYMPLICITY Spyral radiofrequency system (Medtronic), approved on 17 November 2023. Both carry a broad indication, namely the reduction of BP as adjunctive therapy in patients in whom lifestyle modification and antihypertensive medication do not adequately control BP. A third platform, perivascular alcohol injection (Peregrine, Ablative Solutions), remains investigational. Table 1 compares the platforms.

The SYMPLICITY Spyral is a 4-F rapid-exchange catheter bearing four independently controllable electrodes arranged in a helix, delivered through a 6-F, 55 cm guide over a 0.014-inch non-hydrophilic, soft-tipped, supportive guidewire; polymer-jacketed wires should be avoided because of the risk of migration and parenchymal perforation [10]. The helical configuration opposes the electrodes to the vessel wall while leaving the lumen patent so that flowing blood cools the endothelium. Each 60 s run delivers circumferential energy across all four quadrants under an algorithm that continuously monitors impedance and temperature and preferentially heats periadventitial fat. Treatment proceeds from distal to proximal, respecting a margin of at least 5 mm from the renal parenchyma, from any atheroma or stent, from a bifurcation, and from the aorta, with ablations placed every 5 to 6 mm along treatable segments. Selective de-activation of individual electrodes allows for bifurcations to be navigated without overlapping prior treatment zones.

The Paradise system uses a 7-F balloon-based over-the-wire catheter containing a piezoelectric transducer that delivers 360-degree focused ultrasound while circulating sterile water cools the arterial wall [10]. Five balloon sizes are matched to the vessel diameter in a one-to-one fashion. Each emission lasts approximately seven seconds; after which, the balloon is repositioned proximally in 5 mm increments, typically requiring two to five treatments per artery, with the most proximal treatment no closer than 5 mm to the aorta. With the Paradise system, the main renal artery and suitable accessory renal arteries are treated, whereas first-order branches are generally not targeted. Ultrasound denervation generally requires less contrast and shorter fluoroscopy and ablation times than radiofrequency, an advantage in patients with reduced renal function, although early bifurcations are anatomically challenging and variation in vessel diameter may necessitate multiple catheter exchanges [10].

Several periprocedural principles are shared. Antihypertensive medication should be continued according to the outpatient regimen, since withholding multiple agents risks unsafe pre-procedural BP elevation in this population [10]. Moderate sedation with an opioid and benzodiazepine is generally sufficient and is usually managed by the operator, with the caveat that radiofrequency ablation is more painful than ultrasound and requires vigilant re-dosing; analgesia should be administered at least 10 min before energy delivery. Ultrasound-guided common femoral access is standard. Aortography, or pre-procedural computed tomographic or magnetic resonance angiography, excludes anatomic contraindications—including renal artery diameter below 3 mm or above 8 mm, stenosis exceeding 50%, fibromuscular dysplasia, aneurysm, and recent renal stenting—and identifies accessory vessels. Heparin to a therapeutic activated clotting time and intra-arterial nitroglycerin before baseline and completion angiography are routine, whereas antiplatelet therapy is not mandated. A completion angiogram must be reviewed for dissection, perforation, and parenchymal injury.

## 4. The Randomised Evidence Base

### 4.1. First-Generation Trials and the SYMPLICITY HTN-3 Inflection

SYMPLICITY HTN-1 and HTN-2 reported large office BP reductions in open-label designs. SYMPLICITY HTN-3, the first large sham-controlled trial, enrolled 535 patients and showed no significant difference in office or 24 h ambulatory SBP at six months [11]. Subsequent analysis identified a convergence of confounders: a single-electrode catheter treating only the main artery with few ablations, operator inexperience, medication turbulence during run-in and follow-up, unverified adherence, and heterogeneity of the enrolled population.

Two observations from long-term follow-up have proved important. The between-group difference in office and ambulatory SBP widened progressively and became statistically significant at 12, 24, and 36 months, accompanied by a lower antihypertensive medication burden in the denervation arm. Additionally, the resistant hypertension subgroup derived the greatest benefit, with an approximately 15 mm Hg reduction in ambulatory SBP at 36 months [12]. Whether this reflects a genuinely delayed biological effect, differential medication drift after unblinding, or regression phenomena cannot be resolved from an unblinded extension, but it raised the legitimate possibility that six-month endpoints systematically underestimate the treatment effect.

### 4.2. Second-Generation Radiofrequency Trials: The SPYRAL Programme

SPYRAL HTN-OFF MED tested the biological proof of principle in patients not receiving any antihypertensive therapy. The proof-of-concept trial of 80 patients showed between-group differences of 5.0 mm Hg in 24 h SBP and 7.7 mm Hg in office SBP at three months [13], and the pivotal trial of 331 patients confirmed significant reductions of 3.9 mm Hg in 24 h SBP and 6.5 mm Hg in office SBP versus sham [14].

SPYRAL HTN-ON MED extended evaluation to patients taking one to three agents, with adherence verified by witnessed drug intake and urine and plasma toxicology. The proof-of-concept trial of 80 patients demonstrated differences of 7.0 mm Hg in 24 h SBP and 6.6 mm Hg in office SBP at six months [15], with progressive divergence to an adjusted treatment difference of 10 mm Hg in 24 h SBP at 36 months despite comparable medication burden [16]. The powered expansion trial of 337 patients did not meet its primary endpoint of 24 h SBP at six months [17]. Office SBP, nighttime BP, and a win-ratio analysis favoured denervation, and disproportionate medication escalation in the sham arm during the COVID-19 pandemic, which among United States patients reached 37% versus 23%, was the investigators’ principal explanation [17]. This trial must be reported honestly as a negative primary-endpoint study; the supporting analyses are hypothesis-generating rather than confirmatory.

A longer follow-up of the full ON MED cohort is instructive. In the prespecified 24-month analysis, denervation produced significantly greater reductions in both 24 h ambulatory and office SBP than sham despite higher antihypertensive medication use in the sham arm; the change in eGFR was −0.7 ± 11.2 versus −3.1 ± 7.6 mL/min/1.73 m^2^ (*p* = 0.13), and no renal artery stenosis exceeding 70% or renal reintervention occurred during 24 months [18]. Multiple sensitivity analyses, including medication-burden-adjusted comparisons and restriction to patients who remained hypertensive by original trial criteria, consistently favoured denervation [18]. A caveat applies: 74% of sham patients elected to cross over after unblinding at six months, which both attests to patient acceptability and progressively erodes the integrity of the control arm for long-term comparisons.

### 4.3. Ultrasound Denervation: The RADIANCE Programme

RADIANCE-HTN SOLO enrolled 146 patients with combined systolic–diastolic hypertension, withdrawn from up to two agents. At two months, daytime ambulatory SBP fell by 8.5 mm Hg with ultrasound denervation versus 2.2 mm Hg with sham, a between-group difference of 6.3 mm Hg (95% confidence interval 3.1 to 9.4), with no major adverse events [19].

RADIANCE-HTN TRIO enrolled 136 patients with resistant hypertension, all switched before randomisation to a single-pill combination of calcium channel blocker, angiotensin receptor blocker, and thiazide, a rigorous confirmation of true pharmacologic resistance. At two months, the between-group difference in daytime ambulatory SBP was 4.5 mm Hg (95% confidence interval 0.3 to 8.5) [20]. At six months, after blinded medication titration, daytime ambulatory SBP was similar between groups, but the denervation group achieved this with significantly fewer medications, including fewer aldosterone antagonists at 40% versus 60.9% (*p* = 0.02) and lower home SBP, with a between-group difference of 4.3 mm Hg (*p* = 0.03) [21]. This is a clinically important pattern that recurs throughout the field, where BP is titrated to the target and the benefit of denervation manifests as medication sparing rather than as a BP difference.

RADIANCE II randomised 224 patients in a 2:1 allocation and confirmed efficacy in a larger off-medication cohort, with a 6.3 mm Hg greater reduction in daytime ambulatory SBP versus sham (95% confidence interval 3.2 to 9.3), an improvement in six of seven prespecified secondary BP outcomes, and no major adverse events [22]. A three-year follow-up of the treatment arm of RADIANCE-HTN SOLO demonstrated durable office BP reduction with sustained safety, providing the longest published efficacy data for ultrasound denervation [23].

### 4.4. Alcohol-Mediated and Other Platforms

TARGET BP I, enrolling 301 patients taking two to five medications, met its primary endpoint with a between-group difference of 3.2 mm Hg in 24 h ambulatory SBP (*p* = 0.049) without excess major adverse events [24], whereas TARGET BP OFF-MED established safety but did not meet its efficacy endpoint [25]. The magnitude of effect with alcohol-mediated denervation appears to be smaller than with thermal modalities, although no head-to-head comparison exists.

Generalisability beyond Western populations has been addressed by two Chinese sham-controlled trials. Iberis-HTN, enrolling 217 patients treated with a radiofrequency device, demonstrated a six-month between-group difference in 24 h SBP of 9.4 mm Hg (*p* < 0.001) [26], and a six-electrode radiofrequency trial reported substantial office SBP reduction. SMART, enrolling 220 patients, evaluated stimulation-guided mapping and selective ablation and achieved non-inferior office SBP control with a smaller increase in medication burden than sham [27]. Neutral results in REQUIRE, an ultrasound trial conducted in Japan and South Korea, are widely attributed to an unusually large sham-arm reduction, itself probably reflecting improved adherence among enrolled patients with poor baseline adherence [28].

### 4.5. Quantitative Synthesis

**Figure 2:**
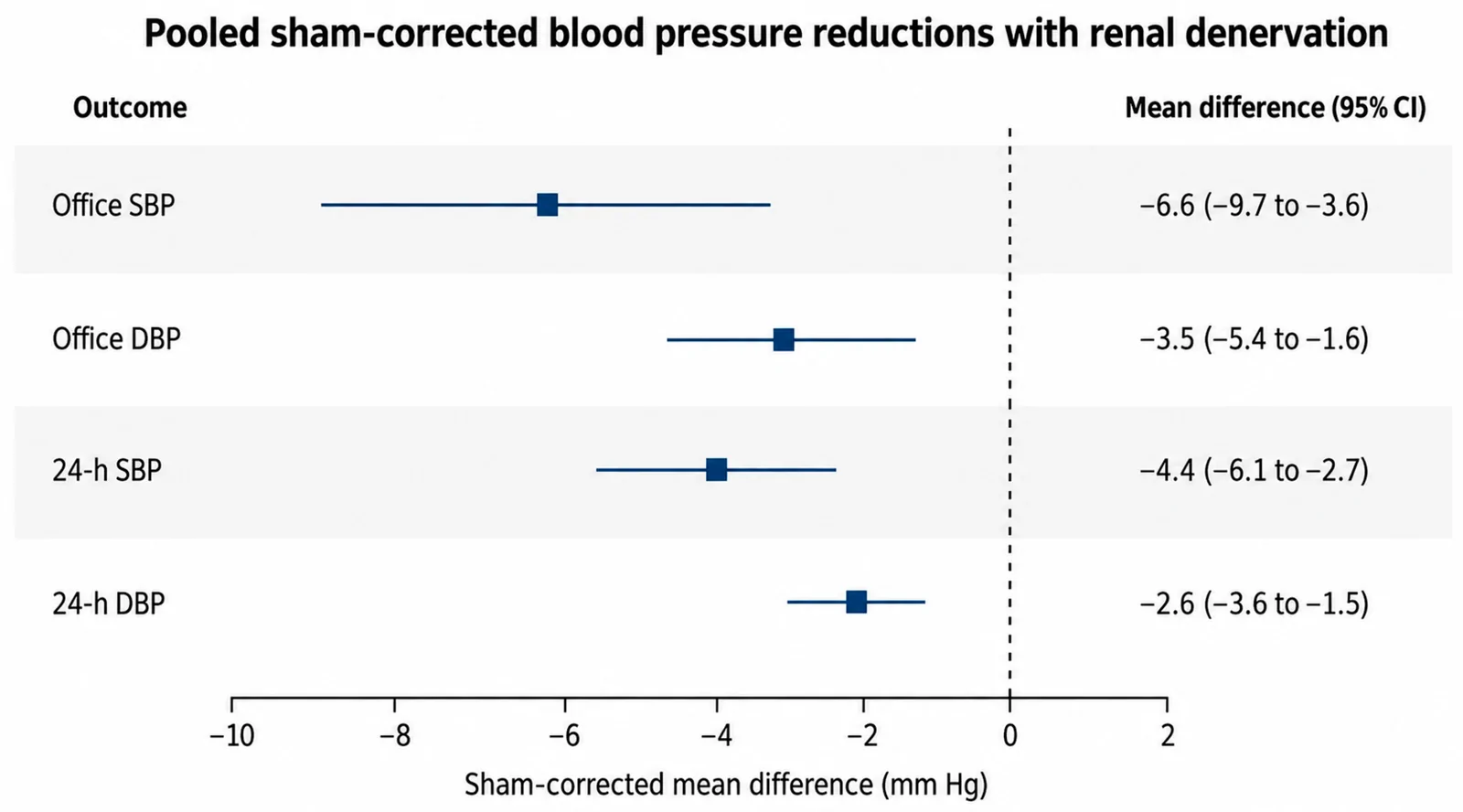
Pooled sham-corrected blood pressure reductions with renal denervation. Forest plot showing pooled sham-corrected mean differences in office and 24 h systolic and diastolic blood pressure after renal denervation. Squares represent pooled mean differences and horizontal lines represent 95% confidence intervals. Negative values favour renal denervation. Subgroup analyses for 24 h systolic blood pressure showed no significant interaction according to medication status or energy modality. 24 h = 24 hours; CI = confidence interval; DBP = diastolic blood pressure; RDN = renal denervation; SBP = systolic blood pressure.

The most methodologically disciplined meta-analysis, restricted to high-quality sham-controlled randomised trials and comprising 13 trials and 2478 patients with median follow-up to the primary endpoint of three months, reported sham-corrected reductions of 6.6 mm Hg in office SBP (95% confidence interval 3.6 to 9.7) and 3.5 mm Hg in office diastolic BP, 4.4 mm Hg in 24 h SBP and 2.6 mm Hg in 24-h diastolic BP, 5.2 mm Hg in daytime SBP, and 4.5 mm Hg in nighttime SBP (95% confidence interval 2.8 to 6.1), with 2.6 mm Hg in nighttime diastolic BP [29]. Critically, there was no significant interaction by concomitant medication—with reductions of 4.8 mm Hg off medication and 4.0 mm Hg on medication (*p* for interaction 0.62)—by device generation or by energy modality, where radiofrequency, ultrasound, and alcohol produced 24 h SBP reductions of 5.2, 4.8, and 2.4 mm Hg, respectively (*p* for interaction 0.18) [29]. Independent meta-analyses have reached concordant conclusions. The pooled sham-corrected reductions across office and ambulatory blood pressure outcomes are summarised in Figure 2.

Two interpretive points matter. These are average effects, whereas the clinically relevant question is distributional rather than one of central tendency, as discussed in Section 7. Furthermore, a 4 to 5 mm Hg reduction in 24 h SBP, sustained over time and superimposed on background therapy, is clinically meaningful given the well-established and consistent relationship between blood pressure reduction and lower cardiovascular risk across epidemiological studies and antihypertensive trials. Table 2 summarises the principal sham-controlled trials.

## 5. Durability and Real-World Evidence

Durability is the central premise of a one-time procedure and is also the hardest property to demonstrate rigorously since it is not ethical to maintain a sham arm untreated for years and virtually all trials mandate medication escalation as a safety measure.

A pooled 36-month analysis of 2137 radiofrequency-treated patients drawn from the Global SYMPLICITY Registry DEFINE cohort, SPYRAL First-in-Human, and both SPYRAL HTN-OFF MED and ON MED trials provides the most comprehensive assessment to date. The baseline office SBP was 163 ± 23 mm Hg on 3.8 ± 2.1 medications. At 36 months, the medication number was 3.5 ± 1.9 and reductions in office and 24 h ambulatory SBP were 18.1 ± 23.4 and 13.3 ± 17.6 mm Hg, respectively (both *p* < 0.0001). Eighty-eight percent of patients met a composite clinical-benefit definition comprising an office SBP reduction of at least 10 mm Hg, an ambulatory SBP reduction of at least 5 mm Hg, or a reduction of at least one medication [30]. A patient-level analysis of 4155 patients across six studies in the same programme showed a steep reduction over the first three months followed by steady further decline through three years and identified higher baseline SBP as the single characteristic most consistently associated with greater response [31].

Real-world data are concordant. Published analyses from the Global SYMPLICITY Registry, encompassing more than 2200 patients with elevated office SBP despite at least three medications, showed office SBP decreases of 12.8 ± 26.2 mm Hg and 24 h ambulatory SBP decreases of 7.2 ± 17.8 mm Hg at six months, with blood pressure lowering being slightly augmented at three years, during which the number of medication classes remained the same for 46.8%, decreased for 31.2%, and increased for 22.0% [32]. A published subgroup analysis of the same registry according to antihypertensive medication use confirmed consistent outcomes across medication strata [33]. Single-centre long-term cohorts have reported sustained BP reduction and reduced medication burden at nine and ten years [34,35].

These figures should be read with discipline. Registry data are uncontrolled and subject to selection bias, regression to the mean, survivor bias as non-responders are lost to follow-up, diminishing white-coat effect, and physician-reported rather than biochemically verified adherence. The defensible conclusion is narrower and still valuable: across published randomised and observational cohorts, there is no signal of attenuation through three years and no reported need for reintervention. The theoretical concern of reinnervation persists since animal studies demonstrate anatomical and partial functional reinnervation while BP reduction is maintained, raising the question of whether the reinnervation that occurs is functionally meaningful [36]. Human data are absent.

## 6. Safety

Safety is the domain in which the evidence is most consistent and most reassuring, and contemporary outcomes are summarised in Table 3.

Across the sham-controlled trials, the overall incidence of vascular complications was 0.5%, with no significant difference between denervation and sham (odds ratio of 1.69, 95% confidence interval from 0.57 to 5.0) [29]. Access-site complications, including haematoma, pseudoaneurysm, and dissection, occur at rates typical of femoral arterial procedures and below 1%. The most common patient-reported adverse effect is procedural and post-procedural pain, reported to persist beyond two days in approximately 12% of patients in earlier meta-analyses [36]. Denervation entails iodinated contrast and radiation exposure, which are not trivial considerations in a population enriched for chronic kidney disease.

Concern regarding renal artery injury has not been borne out. The annualised incidence of renal artery stenting after radiofrequency denervation is approximately 0.2%, comparable to the background rate of atherosclerotic renal artery stenosis in hypertensive populations, and no significant difference from sham has been observed (odds ratio of 1.50, 95% confidence interval from 0.06 to 36.97) [29,36]. Systematic imaging follow-up has shown no association with vascular injury or de novo stenosis up to 36 months, and in SPYRAL HTN-ON MED, no stenosis exceeding 70% and no reintervention occurred up to 24 months [18]. Routine post-procedural imaging is not mandated, although duplex ultrasound, computed tomographic angiography, or magnetic resonance angiography is reasonable when clinically indicated.

Renal function is likewise preserved. In the pooled sham-controlled analysis, the change in eGFR did not differ between denervation, at −0.75 mL/min/1.73 m^2^, and sham, at −0.62 mL/min/1.73 m^2^ (*p* for interaction 0.84) [29]. The published registry data show that the annual eGFR decline in patients with chronic kidney disease is not significantly different from that in patients without and is comparable to the age-expected decline [33]. Rates of end-stage renal disease and of a 50% or greater rise in creatinine in registry populations are consistent with the natural history of this comorbid population rather than with procedural harm.

## 7. The Unsolved Problem of Response Heterogeneity

The single most important limitation of RDN is not efficacy or safety but prediction. The average treatment effect conceals wide inter-individual variability, and up to 30% of treated patients derive little or no BP benefit [10]. In the pooled 36-month analysis, 12% of patients did not meet any component of the composite clinical-benefit definition despite undergoing an invasive procedure [30]. These are not failures of the therapy so much as a mismatch between a broad regulatory indication and an incompletely characterised responder phenotype.

Aside from higher baseline SBP, no reliable pre-procedural predictor has been identified [31]. Factors reported to attenuate response include lower baseline BP, greater arterial stiffness, and anatomic variants such as untreated accessory arteries [36]. It is worth noting that pharmacotherapy suffers from the same limitation since the response to any given antihypertensive agent is similarly unpredictable, but the asymmetry in cost, invasiveness, and irreversibility makes the problem more consequential for a device.

Unlike percutaneous coronary intervention, which offers an angiographic and physiological result; transcatheter aortic valve replacement, which offers a gradient; or pulmonary vein isolation, which offers entrance and exit block, renal denervation has no intraprocedural marker of technical success. The operator cannot know at the end of the case whether the nerves have been adequately interrupted. Developing such a marker, whether through stimulation-based mapping, biomarkers of sympathetic activity, or the imaging of nerve distribution, is arguably the highest-value research priority in the field.

## 8. Blood Pressure Reduction and Cardiovascular Outcomes

No adequately powered randomised trial has evaluated the effect of RDN on major adverse cardiovascular events. The obstacles are formidable and include high crossover rates after unblinding, the need for prolonged follow-up, confounding by adherence and lifestyle drift, low residual event rates in contemporary hypertension trials, and cost.

Two lines of indirect evidence exist. First, an analysis of the Global SYMPLICITY Registry showed that greater time spent within the target SBP range after denervation was associated with fewer major cardiovascular events, although this is an observational association within a treated cohort rather than a randomised comparison [34]. Second, epidemiological extrapolation from the established relationship between sustained BP reduction and cardiovascular risk supports benefit [37]. Notably, in the published meta-analysis of sham-controlled trials, there was no significant difference in hypertensive crisis (odds ratio of 0.65, 95% confidence interval from 0.30 to 1.38) or all-cause death (odds ratio of 1.76, 95% confidence interval from 0.34 to 9.20) between the denervation and sham groups, but these analyses were underpowered for clinical-event endpoints [29].

Programmes are beginning to address the gap in populations with concentrated residual risk. The EMBRACE trial randomises patients with uncontrolled hypertension and multivessel coronary artery disease to radiofrequency denervation added to staged percutaneous coronary intervention versus staged intervention alone, a design that deliberately selects a population in whom event rates are high enough to make an outcome trial feasible. Although renal denervation-specific randomised cardiovascular outcome data remain limited, the sustained reduction in BP achieved with the procedure would be expected to translate into cardiovascular benefit based on the well-established relationship between BP lowering and cardiovascular risk reduction.

## 9. Special Populations

### 9.1. Chronic Kidney Disease

Chronic kidney disease and hypertension are bidirectionally linked through sympathetic overactivity, making chronic kidney disease a mechanistically attractive indication. Experimental data provide additional mechanistic support for this concept. In models of obstructive nephropathy and renal ischemia–reperfusion injury, renal sympathetic and sensory nerve signalling promoted inflammatory and profibrotic pathways, whereas renal denervation attenuated leukocyte infiltration, profibrotic signalling, and tubulointerstitial fibrosis. However, patients with eGFR below 40–45 mL/min/1.73 m^2^ were excluded from the pivotal sham-controlled trials. Accordingly, European guidelines recommend renal denervation only in patients with an eGFR of at least 40 mL/min/1.73 m^2^, whereas United States recommendations use a threshold of 30 [36,37,38,39,40].

The available evidence is encouraging but remains incomplete. Registry analyses and dedicated subgroup studies consistently demonstrate BP reductions comparable to those observed in patients without chronic kidney disease, without evidence of accelerated renal function decline through three years of follow-up [33,41]. Small sham-controlled and observational studies similarly suggest preserved eGFR and possible reductions in albuminuria, although these findings remain susceptible to bias. Experience in end-stage renal disease is limited to small case series demonstrating procedural feasibility in selected patients. Overall, renal denervation appears safe in chronic kidney disease stage 3 and probably stage 3b, but nephroprotection remains unproven and requires dedicated randomised trials with renal endpoints.

### 9.2. Diabetes, Isolated Systolic Hypertension, and Prior Stroke

Subgroup analyses of sham-controlled trials suggest that the BP-lowering efficacy of renal denervation is broadly consistent across patient populations, including those with diabetes and elevated cardiovascular risk, without evidence of differential safety signals [29,33]. However, the evidence remains limited in isolated systolic hypertension, and current device labelling does not establish safety or efficacy in this population. Ongoing post-approval studies, including SPYRAL AFFIRM, are expected to clarify outcomes in higher-risk subgroups.

### 9.3. Heart Failure

The biological rationale for renal denervation in heart failure is compelling, given the central role of sympathetic and renin–angiotensin–aldosterone activation in disease progression. Clinical evidence, however, remains limited. Small, randomised studies in heart failure with reduced ejection fraction have reported mixed findings, with meta-analyses suggesting improvements in functional capacity, left ventricular function, and natriuretic peptide levels despite important methodological limitations [6].

Heart failure with preserved ejection fraction may represent an even more attractive target because of its close association with hypertension. Although mechanistic studies have suggested favourable haemodynamic effects, the RDT-PEF trial failed to meet its primary endpoint [6]. At present, renal denervation should still be regarded as investigational in heart failure irrespective of phenotype.

### 9.4. Atrial Fibrillation

Hypertension is the dominant modifiable driver of atrial fibrillation, and the rationale for adjunctive denervation at the time of ablation is sound. ERADICATE-AF randomised patients with hypertension and paroxysmal atrial fibrillation to pulmonary vein isolation with or without radiofrequency denervation and found a 43% reduction in the hazard of atrial arrhythmia recurrence at 12 months [42]. A subsequent randomised study missed its primary efficacy endpoint but significantly reduced atrial arrhythmia burden and antiarrhythmic drug requirement, a sham-controlled trial in resistant hypertension reported a 60% reduction in the hazard of subclinical atrial fibrillation at two years, and a pilot study of adjunctive ultrasound denervation found numerically but not statistically greater freedom from atrial arrhythmia at 12 months [6]. This is a promising but unconfirmed indication.

## 10. Guidelines, Regulation, and Reimbursement in 2026

### 10.1. Guideline Convergence

Three major guidelines now address renal denervation, and their apparent divergence in recommendation class conceals substantial agreement, as summarised in Table 4. The 2023 ESH guidelines recommend denervation for selected patients with uncontrolled or resistant hypertension and in those with drug intolerance, provided the procedures are performed in experienced centres [38]. The 2024 ESC guidelines similarly support the consideration of RDN in patients with uncontrolled hypertension at increased cardiovascular risk, including those with medication non-adherence or intolerance, emphasising multidisciplinary assessment and specialised centres [39]. The 2025 AHA/ACC multisociety guideline assigns a Class 2b recommendation for adults with resistant or uncontrolled hypertension who prefer a non-pharmacologic approach or have difficulty adhering to therapy, representing the first formal recognition of RDN in a United States cardiovascular society guideline following the 2024 AHA scientific statement [36,40].

All three converge on four indications: uncontrolled hypertension despite pharmacotherapy, resistant hypertension confirmed by ambulatory monitoring, drug intolerance, and informed patient preference. Shared decision-making is a consistent recommendation, whereas the principal differences relate to eGFR thresholds and the emphasis is placed on centre experience.

### 10.2. The United States National Coverage Determination

For two years after regulatory approval, United States adoption was constrained less by evidence than by reimbursement, as both approved systems were assigned Category III procedural codes without relative value units. This changed on 28 October 2025, when the Centres for Medicare and Medicaid Services issued National Coverage Determination 20.40 for renal denervation in uncontrolled hypertension [43].

The coverage determination largely codifies the principles already advocated by hypertension specialists. Eligibility requires documented uncontrolled hypertension despite lifestyle modification and stable, maximally tolerated guideline-directed medical therapy; confirmation by ambulatory or home BP monitoring; assessment of adherence; exclusion of secondary hypertension; and the absence of contraindications according to device labelling [44]. Procedures must be performed by appropriately trained operators within multidisciplinary hypertension programmes, with continued participation in a coverage-with-evidence-development programme [44]. Clinicians should note that the determination applies to Medicare beneficiaries and does not bind commercial or Medicaid payers, which maintain independent reimbursement policies.

Formal cost-effectiveness analyses have consistently reported favourable incremental cost-effectiveness ratios, including approximately USD 32,700 per quality-adjusted life-year for radiofrequency denervation in the United States setting [44]. These analyses remain dependent on modelled cardiovascular benefit extrapolated from BP reduction rather than demonstrated outcome data and most have been industry sponsored.

### 10.3. Building and Running a Programme

The coverage determination reinforces an important principle: renal denervation is a programme rather than a procedure. A successful programme requires close collaboration between an interventionalist, a hypertension specialist responsible for medication optimisation and exclusion of secondary causes, and a nurse navigator coordinating patient selection, ambulatory monitoring, scheduling, and follow-up [45]. Clear communication with referring physicians and realistic patient counselling regarding the expected magnitude and timing of BP reduction, the continued need for medication, and the possibility of non-response are essential.

**Figure 3:**
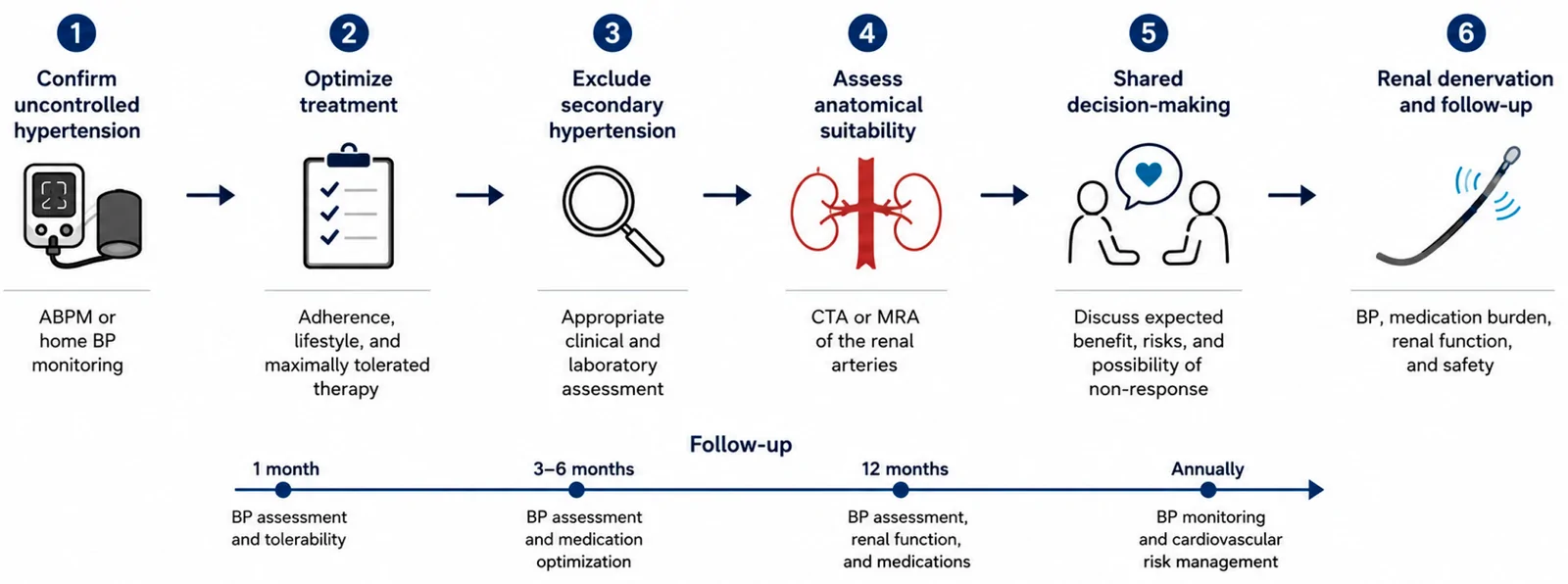
Contemporary patient pathway for catheter-based renal denervation. Simplified clinical pathway for renal denervation, from confirmation of uncontrolled hypertension and treatment optimisation to exclusion of secondary causes, anatomical assessment, shared decision-making, the procedure, and structured follow-up. Follow-up includes reassessment of blood pressure, medication burden, renal function, tolerability, and cardiovascular risk. ABPM = ambulatory blood pressure monitoring; BP = blood pressure; CTA = computed tomography angiography; MRA = magnetic resonance angiography; RDN = renal denervation.

Patient preference is an important component of shared decision-making. Across international studies, approximately 30% of patients receiving antihypertensive medication and 35% to 40% of those not taking medication express a preference for RDN over additional pharmacotherapy [46]. Although physicians tend to prioritise patients with the highest BP and medication burden, patient interest is driven more by treatment burden and adverse effects than by BP severity itself, with physician recommendation consistently emerging as the strongest determinant of treatment acceptance [43]. A contemporary clinical pathway encompassing patient selection, pre-procedural assessment, shared decision-making, renal denervation, and structured follow-up is presented in Figure 3.

## 11. Emerging Technologies and Future Directions

Efforts to achieve precision denervation follow two complementary strategies. Stimulation-guided mapping and selective ablation aim to identify and target sympathetically active regions, an approach validated against sham in the SMART trial and currently under evaluation in a real-world registry of approximately 1000 patients [27]. Anatomically optimised protocols offer a simpler alternative: in the SPYRAL DYSTAL pilot, treatment confined to the distal main renal artery and primary branches reduced procedure time, contrast use, and the number of ablations while achieving favourable 12-month ambulatory BP reduction [47]. Given that contrast exposure remains an important procedural limitation, particularly in patients with chronic kidney disease, these findings warrant confirmation in larger trials.

Several new platforms and access strategies are also under evaluation, including non-occlusive intravascular ultrasound systems, alcohol-mediated denervation, radial-access platforms, and extravascular or non-arterial approaches. Although these technologies seek to improve procedural simplicity, safety, or efficacy, they remain investigational and require sham-controlled validation. Ongoing and recently initiated studies are summarised in Table 5.

Sympathetic overactivity is not confined to the renal axis, and multi-organ denervation represents a conceptually distinct extension. Combined renal and common hepatic artery denervation has shown encouraging preclinical results and is currently undergoing first-in-human evaluation [48]. If successful, this strategy could extend catheter-based denervation beyond hypertension toward broader cardiometabolic disease [49].

Any assessment of the future role of renal denervation must also account for concurrent pharmacological innovation. In May 2026, the US Food and Drug Administration approved baxdrostat, an oral aldosterone synthase inhibitor, as add-on therapy for adults with inadequately controlled hypertension, following the phase III BaxHTN trial, which demonstrated significant placebo-corrected reductions in seated SBP [50]. Together with agents such as aprocitentan [41], these therapies reinforce that the future is unlikely to be one of competition between drugs and devices, but of patient phenotyping and treatment sequencing. Comparative trials between contemporary pharmacotherapy and renal denervation are now needed.

## 12. Research Agenda

Five priorities emerge. First, the development of an intraprocedural endpoint is essential since renal denervation remains the only mature cardiovascular intervention performed without confirmation of procedural success; stimulation-based mapping, biomarkers, and nerve imaging all warrant further investigation. Second, responder phenotyping requires prospective validation of candidate predictors, including baseline SBP, arterial stiffness, plasma renin activity, nocturnal BP pattern, and renal artery anatomy. Third, cardiovascular outcome trials remain a priority, with pragmatic registry-based designs likely to be more feasible than conventional megatrials. Fourth, comparative and combination studies against contemporary antihypertensive therapies, including aldosterone synthase inhibitors, are needed. Finally, dedicated randomised trials in patients with stages 3b–4 chronic kidney disease should determine whether renal denervation confers true nephroprotection beyond BP reduction.

Two methodological priorities should accompany these efforts. The standardisation of trial endpoints and follow-up would facilitate meaningful comparisons across devices, while greater support for investigator-initiated and publicly funded research would help balance an evidence base that remains largely industry-sponsored.

## 13. Conclusions

Renal denervation in 2026 is a therapy of established, modest, and durable efficacy, with a safety profile among the best characterised of any interventional cardiovascular procedure. Thirteen sham-controlled randomised trials consistently demonstrated sham-corrected reductions of approximately 4 to 5 mm Hg in 24 h ambulatory SBP and 6 to 7 mm Hg in office SBP, irrespective of energy modality or background pharmacotherapy. Three-year randomised and real-world data show no evidence of attenuation, and the therapy is now incorporated into European and—for the first time—United States guidelines.

Important uncertainties remain. Cardiovascular outcome benefit is inferred rather than demonstrated, a substantial minority of patients do not respond, and no intraprocedural marker reliably confirms successful denervation. At the same time, new antihypertensive therapies are reshaping the treatment landscape, reinforcing the need for careful patient selection rather than competition between pharmacological and device-based approaches.

The appropriate posture is therefore neither the enthusiasm of 2011 nor the dismissal of 2014, but disciplined integration. Renal denervation belongs in the armamentarium of hypertension care, offered within a multidisciplinary programme to appropriately selected patients after shared decision-making that presents both the expected benefit and the possibility of non-response. Delivered in that way, it will serve patients well; delivered as a volume-driven procedure detached from comprehensive hypertension care, it risks repeating the mistakes of the first generation.

## Figures and Tables

**Table 1:** Approved and investigational catheter-based renal denervation platforms.

Feature	SYMPLICITY Spyral	Paradise	Peregrine	Non-Occlusive Ultrasound
Energy or mechanism	Radiofrequency	Focused ultrasound, balloon-based, water-cooled	Perivascular dehydrated alcohol	Intravascular ultrasound, blood-cooled
Regulatory status	Approved November 2023	Approved November 2023	Investigational	Investigational
Sheath size	6-F guide, 4-F catheter	7-F	7-F	4-F, radial-capable
Design	Helical, four independent electrodes	Balloon-mounted piezoelectric transducer, five sizes	Three microneedles at 120 degrees	Non-balloon transducer
Target vessels	Main, first-order branches, accessory (3–8 mm)	Main and accessory (3–8 mm); not branches	Main and accessory	Main
Energy delivery	60 s per run; 20–48 ablations per patient	Two to three emissions of ~7 s per artery	0.6 mL alcohol per main artery	Continuous, non-occlusive
Cooling	Endogenous blood flow	Circulating sterile water	Not applicable	Endogenous blood flow
Key pivotal evidence	SPYRAL HTN-OFF MED and ON MED	RADIANCE-HTN SOLO and TRIO; RADIANCE II	TARGET BP I	Randomised trial ongoing

Comparison of catheter-based renal denervation platforms according to mechanism, catheter profile, target vessels, energy delivery, cooling method, regulatory status, and pivotal clinical evidence. F = French; RDN = renal denervation.

**Table 2:** Principal randomised sham-controlled trials of renal denervation.

Trial (Year)	n	Modality	Population and Medication Status	Primary Endpoint	Result Versus Sham	Primary Endpoint Met
SYMPLICITY HTN-3 (2014)	535	Radiofrequency, first-generation	Resistant hypertension, on medication	Office SBP, 6 months	−2.4 mm Hg, NS	No
SPYRAL HTN-OFF MED pilot (2017)	80	Radiofrequency	Off medication	24-h SBP, 3 months	−5.0 mm Hg	Yes
SPYRAL HTN-OFF MED Pivotal (2020)	331	Radiofrequency	Off medication	24 h SBP, 3 months	−3.9 mm Hg	Yes
SPYRAL HTN-ON MED pilot (2018)	80	Radiofrequency	One to three medications	24 h SBP, 6 months	−7.0 mm Hg	Yes
SPYRAL HTN-ON MED Expansion (2023)	337	Radiofrequency	One to three medications	24 h SBP, 6 months	NS; office SBP, nighttime BP and win ratio favoured RDN	No
RADIANCE-HTN SOLO (2018)	146	Ultrasound	Off medication, up to two withdrawn	Daytime ambulatory SBP, 2 months	−6.3 mm Hg	Yes
RADIANCE-HTN TRIO (2021)	136	Ultrasound	Resistant hypertension on triple single-pill combination	Daytime ambulatory SBP, 2 months	−4.5 mm Hg	Yes
RADIANCE II (2023)	224	Ultrasound	Off medication	Daytime ambulatory SBP, 2 months	−6.3 mm Hg	Yes
REQUIRE (2022)	143	Ultrasound	Resistant hypertension	24 h SBP, 3 months	NS; large sham response	No
TARGET BP OFF-MED (2023)	106	Alcohol	Off medication	24 h SBP	NS	No
TARGET BP I (2024)	301	Alcohol	Two to five medications	24 h SBP, 3 months	−3.2 mm Hg (*p* = 0.049)	Yes
Iberis-HTN (2024)	217	Radiofrequency	Uncontrolled hypertension, China	24 h SBP, 6 months	−9.4 mm Hg	Yes
SMART (2024)	220	Radiofrequency, mapping-guided	Uncontrolled hypertension, China	Office SBP control, 6 months	Non-inferior control with less medication escalation	Yes

Principal randomised sham-controlled trials evaluating the efficacy of renal denervation in patients with uncontrolled or resistant hypertension. Effect estimates represent between-group differences at the prespecified primary endpoint. NS = not significant; RDN = renal denervation; SBP = systolic blood pressure; 24 h = 24 hours.

**Table 3:** Contemporary safety outcomes of renal denervation.

Domain	Finding
Vascular complications	0.5% across sham-controlled trials; no difference versus sham (OR 1.69, 95% CI 0.57–5.0)
Access-site complications	Below 1%; comparable to other femoral arterial procedures
Renal artery stenosis	Approximately 0.2% per year requiring stenting, comparable to background rate; OR versus sham 1.50 (95% CI 0.06–36.97)
Renal artery stenosis above 70% or reintervention	None through 24 and 36 months in SPYRAL HTN-ON MED
Imaging-verified vascular injury	None through 36 months on systematic imaging follow-up
Change in eGFR	−0.75 (RDN) versus −0.62 (sham) mL/min/1.73 m^2^; *p* for interaction 0.84
eGFR trajectory in chronic kidney disease	Annual decline comparable to age-expected and to patients without chronic kidney disease
Hypertensive crisis	OR 0.65 (95% CI 0.30–1.38)
All-cause death	OR 1.76 (95% CI 0.34–9.20)
Composite safety endpoint at 36 months	3.3% (RDN) versus 2.7% (sham); *p* > 0.99
Procedural pain	Most common adverse effect; persisting beyond 2 days in approximately 12%

Summary of vascular, renal, procedural, and clinical safety outcomes reported in contemporary randomised trials and observational cohorts of renal denervation. CI = confidence interval; eGFR = estimated glomerular filtration rate; OR = odds ratio; RDN = renal denervation.

**Table 4:** Guideline and coverage positions on renal denervation, 2023–2026.

	ESH 2023	ESC 2024	AHA/ACC 2025	CMS NCD 20.40 (2025)
Recommendation class	Class II	Class IIb	Class 2b, level of evidence B-R	Coverage with evidence development
Uncontrolled hypertension despite combination therapy	Yes	Yes, including fewer than three drugs if high cardiovascular risk	Yes	Yes, at or above 140/90 mm Hg
Resistant hypertension	Yes	Yes, regimen must include thiazide or thiazide-like diuretic	Yes	Not explicitly required
Drug intolerance or non-adherence	Yes, serious side effects and poor quality of life	Yes	Yes, difficulty adhering	Adherence assessment required
Patient preference	Required consideration	Required consideration	Required consideration	Documented shared decision-making
eGFR threshold	≥40 mL/min/1.73 m^2^	≥40 mL/min/1.73 m^2^	≥30 mL/min/1.73 m^2^ per US statements	Per device labelling
Blood pressure confirmation	ABPM, excluding white-coat effect	ABPM	ABPM or home	ABPM or serial home monitoring, mandatory
Secondary hypertension exclusion	Yes	Yes	Yes	Minimum of primary aldosteronism, obstructive sleep apnoea, drug- or alcohol-induced
Centre requirement	Experienced specialised centres	Medium-to-high volume, multidisciplinary	Multidisciplinary team	Hypertension programme, navigator, imaging, appropriate suite
Operator requirement	Not specified	Not specified	Not specified	Proctoring and case minimums specified

Comparison of European and United States guideline recommendations and Medicare coverage requirements regarding patient selection, blood pressure confirmation, renal function, secondary hypertension assessment, shared decision-making, and centre requirements. ABPM = ambulatory blood pressure monitoring; ACC = American College of Cardiology; AHA = American Heart Association; CMS = Centres for Medicare and Medicaid Services; eGFR = estimated glomerular filtration rate; ESC = European Society of Cardiology; ESH = European Society of Hypertension; NCD = national coverage determination.

**Table 5:** Selected ongoing and recently initiated studies of renal denervation.

Study	Design	Question Addressed
SPYRAL AFFIRM	Prospective, single-arm, international post-approval	Long-term safety, efficacy, and durability in routine practice, including high-risk phenotypes
SPYRAL CARE	Claims-based coverage-with-evidence-development study	Real-world outcomes in Medicare beneficiaries under NCD 20.40
National renal denervation registry	National outcomes registry	Long-term safety and effectiveness across United States centres
THRIVE	Randomised, sham-controlled	Non-occlusive intravascular ultrasound denervation off medication
RADAR	Randomised, sham-controlled	Alcohol-mediated denervation off medication, higher entry threshold and dose
SMART-RW	Prospective registry, approximately 1000 patients	Mapping-guided selective denervation in real-world practice
SPYRAL GEMINI	First-in-human, single-arm	Combined renal and common hepatic artery denervation
EMBRACE	Randomised	Denervation added to staged percutaneous coronary intervention versus staged intervention alone in uncontrolled hypertension with multivessel coronary disease
RDN-ADPKD	Randomised	Ultrasound denervation in autosomal dominant polycystic kidney disease, with 3-year renal endpoints

Selected studies evaluating long-term effectiveness, novel energy platforms, mapping-guided ablation, combined-organ denervation, coverage-based real-world outcomes, and renal denervation in high-risk populations. ADPKD = autosomal dominant polycystic kidney disease; PCI = percutaneous coronary intervention; RDN = renal denervation; RW = real-world.

## Data Availability

No new data were created or analyzed in this study. Data sharing is not applicable to this article.

## Abbreviations

The following abbreviations are used in this manuscript:

ABPM	ambulatory blood pressure monitoring
BP	blood pressure
CED	coverage with evidence development
CKD	chronic kidney disease
CMS	Centres for Medicare and Medicaid Services
DBP	diastolic blood pressure
eGFR	estimated glomerular filtration rate
ESC	European Society of Cardiology
ESH	European Society of Hypertension
HFpEF	heart failure with preserved ejection fraction
NCD	national coverage determination
RDN	renal denervation
SBP	systolic blood pressure
